# NTP Draft Brief on DEHP

**DOI:** 10.1289/ehp.114-a580

**Published:** 2006-10

**Authors:** Julia R. Barrett

Questions about the safety of the plasticizer di(2-ethylhexyl) phthalate (DEHP), particularly in regards to exposure during medical procedures such as transfusions, have swirled for decades, but especially in the last several years, given growing concerns about endocrine disruption. In October 2005, an independent panel of experts convened by the National Toxicology Program Center for the Evaluation of Risks to Human Reproduction (NTP-CERHR) sought to take stock of what is known and identify critical research needs regarding human exposure to DEHP, in particular its potential reproductive and developmental toxicity. Now that the independent experts have had their say, the NTP is weighing in with its interpretation.

Based on the expert panel’s report, comments from stakeholders and peer reviewers, and new information published since the experts’ meeting, the NTP released a draft brief in May 2006 about DEHP exposure and toxicity. With peer review completed in late August, the brief is now being finalized and will be added to the forthcoming NTP-CERHR monograph *The Potential Human Reproductive and Developmental Effects of DEHP*.

This monograph will comprise the CERHR expert panel report, a list of the panel experts, all public comments made about the report, and the NTP brief on DEHP. Although the brief summarizes what the expert report says, it is more than just an executive summary—it represents the NTP’s view of the various public and peer-review comments and additional research studies received since the report was prepared.

The 2005 expert panel meeting marks the first time the CERHR has had a compound re-evaluated; a previous evaluation was published in 2000. The need for another just five years later underscores the intensity with which DEHP is being investigated.

“[Assessing] DEHP again shows that the CERHR process is evergreen,” says Paul Foster, deputy director of the NTP-CERHR. “This is the first time that CERHR has gone back and said there’s now been a significant amount of water that’s gone under the bridge, and we should go back and re-evaluate to see whether or not any of our original conclusions have changed.”

According to Foster, the brief distills the intricate and detailed scientific knowledge of the monograph into information that educated laypeople can use to put concerns about the potential for DEHP toxicity into perspective.

## Hard Science on a Softener

DEHP is an oily chemical that confers flexibility to rigid polyvinyl chloride plastic. DEHP-softened plastic appears in numerous products, including building materials, food packaging, and medical devices. Because DEHP does not form tight chemical bonds with the plastic, some amount can leach out, and the compound has been detected in packaged foods, indoor air, household dust, and various substances and paraphernalia associated with medical treatment (such as bagged blood and tubing).

DEHP has induced reproductive and developmental problems in male rodents, but there are scant and uncertain data for effects in humans. It is known, however, that low-level human exposure is widespread and that certain populations are more highly exposed. For example, according to the draft brief, newborns and infants undergoing particular medical procedures may have 100 to 1,000 times the exposure experienced by the general population.

Because animal studies indicate that the developing male reproductive system is especially vulnerable to adverse DEHP-associated effects, the expert panel, in its 2005 report, attached “serious concern” to critically ill male newborns and infants receiving prolonged medical treatment. The NTP concurred in its draft brief and also agreed that concern is warranted for male infants younger than 1 year and for the sons of women who underwent certain medical procedures while pregnant. Less concern was attached to low-level exposures *in utero* or after the first year of life, and there was minimal concern for adverse effects from typical background exposures.

## Fairness and Balance

The draft brief is generally deemed fair by both scientists and stakeholders. “To me, it seemed to be very fair based on the discussions and deliberations at the expert review committee,” says Foster. The American Chemistry Council’s Phthalate Esters Panel considered both the brief and the expert panel’s report “fair, but very conservative,” says Marian Stanley, the panel’s senior director. “We’re certainly pleased to see that the areas of concern have been lowered [from the 2000 report] for a couple of cases [children older than a year and pregnant or lactating women]. We think that’s justified.”

The Phthalate Esters Panel disagrees, however, with the NTP’s level of concern about DEHP exposure among newborns and infants. “DEHP medical devices have been used for better than fifty years, and there hasn’t been any verified evidence of harm to humans. We don’t believe that there needs to be as much concern for critically ill neonates because, as the FDA has said [in a July 2002 Public Health Notification], the treatment outweighs any risks from exposure to DEHP,” says Stanley.

Health Care Without Harm (HCWH), a coalition of health and environmental groups that, among other issues, advocates replacing DEHP-containing medical devices with alternatives, was satisfied with the NTP’s position. “We don’t have any quibbles with [who was determined to be] medically exposed, because the panel has expressed serious concern about that and we agree with that,” says Ted Schettler, science director of the Science and Environmental Health Network, on behalf of HCWH.

Schettler avoids defining a level of concern for DEHP exposure of pregnant and lactating women: “We remain concerned about that group of women. Whether we want to say it’s some concern or more than that, we think it should be emphasized that in the general population, pregnant and lactating women are exposed not only to DEHP but also to other phthalates that work through a common toxicological mechanism. The committee wasn’t charged with addressing aggregate exposures to multiple phthalates, but that’s the real world.”

## Outstanding Questions

The question of aggregate exposures is, of course, a scientific dilemma facing the risk assessment community at large, not just the CERHR. Still, says Foster, “I think one of our weaknesses is that we do these evaluations based on single chemicals. I think what’s emerging from a lot of the exposure information that’s being published, mainly from the CDC but also from others in Europe, is that the population at large is exposed to multiple phthalates. We have not really devised an appropriate method yet for how we handle that and put it into a risk context.” He adds that the CERHR system will need to be adapted as new, appropriate methodologies become available.

Another notable challenge is extrapolating results from animal studies to human health. “I think we’re going to continue seeing much more research trying to tease out and figure out if the effects we see in rodents are relevant to humans. This isn’t cut-and-dried research,” says Stanley.

Research with nonhuman primates hasn’t proven any simpler and represents one of the more contentious reactions to the brief. According to Schettler, there’s disagreement about whether nonhuman primates, specifically marmosets, are less vulnerable to DEHP than rodents, as suggested by research published in the October 2005 issue of *Birth Defects Research B: Developmental and Reproductive Toxicology*. Industry-sponsored research indicates that marmosets are a good study model for predicting toxicity in humans, but the October 2005 study, published just as the expert panel meeting concluded, questions that belief, and the debate has not yet been satisfactorily resolved.

Also unresolved are questions about the metabolism of DEHP and its mechanisms of toxicity. The limited epidemiologic data reviewed in the draft brief raise questions that cannot be answered yet. Research is ongoing in all areas, however. “The science is still moving forward; the science is still being created,” says Stanley. “As new techniques become available, there at some point is going to come where science suddenly takes a quantum leap and we can start understanding a lot more.”

## Figures and Tables

**Figure:**
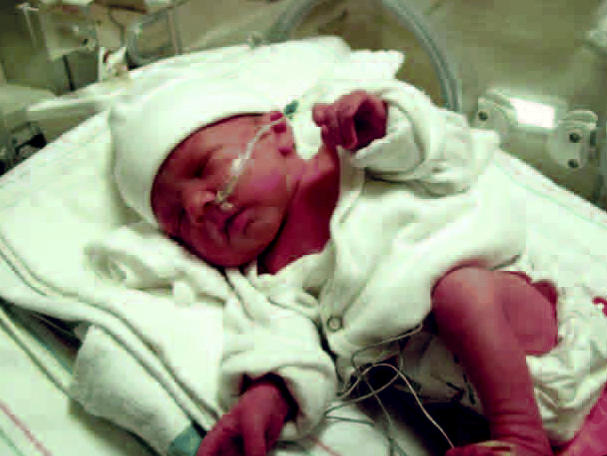
Harming while healing? Concerns about potential reproductive effects of exposure to the plasticizer DEHP, including those to infants from uses in medical tubing and other equipment, prompted a new examination of the available health data by the NTP.

